# Protocol for a systematic review to understand the long-term mental-health effects of influenza pandemics in the pre-COVID-19 era

**DOI:** 10.1177/14034948231217362

**Published:** 2023-12-28

**Authors:** Jessica L. Dimka, Benjamin M. Schneider, Svenn-Erik Mamelund

**Affiliations:** Centre for Research on Pandemics & Society (PANSOC), Oslo Metropolitan University, Norway

**Keywords:** Pandemics, mental health, long-term health consequences, influenza

## Abstract

**Aims:**

This protocol describes a forthcoming systematic review of the question: ‘What are the long-term effects of historical influenza pandemics on mental health, resulting either from illness itself or the social or economic effects of pandemics and public health responses?’

**Methods:**

We will review studies that investigate associations between influenza pandemics and long-term mental-health impacts. Following the PICO framework, populations (P) may include those with and without pre-existing mental-health symptoms or conditions. Intervention (I) is exposure to an influenza pandemic during the study period encompassing five pandemics (1889–2009). Comparators or controls (C) are not applicable. The review will address outcomes (O) of mental-health morbidity from direct infection and/or related circumstances, including, for example, receiving a disability pension, institutionalisation and/or death.

**Results:**

Due to societal disruptions, illness and bereavement during pandemics, many people are likely to be affected in myriad ways. Therefore, investigation into mental-health consequences should not be restricted by risk group or diagnosis. To our knowledge, this protocol and forthcoming systematic review are the first to include studies for broad populations and multiple measures of mental-health morbidity. The historical perspective and comparison of pandemics with varying severity but assumed similar causative pathogens also enable insights into the consistency of long-term consequences across pandemics.

**Conclusions:**

**Pandemics likely produce long-term mental-health impacts with relevance for social, health and economic planning. The systematic review based on this protocol will complement other evidence on pandemic impacts and help policymakers incorporate relevant interventions.**

## Introduction

Health outcomes of infectious disease pandemics are not simply death or complete recovery. Long-term consequences, including mental-health impacts, are also likely and thus have substantial implications for social, health and economic resources and planning. In addition to potential long-term sequelae of infection, mental-health impacts may include new or worsened mental-health symptoms (e.g. depression or anxiety) in infected individuals as well as caretakers, friends and family [1]. As we transition to a post COVID-19-era, there is a need to review long-term mental-health consequences to understand the potential burden and duration of mental-health consequences and to ascertain whether these consequences are specific to or vary by causative agents. In this protocol for a forthcoming systematic review, we address the research question: ‘What are the long-term effects of influenza pandemics on mental health, resulting either from illness itself or the social or economic effects of pandemics and public health responses?’

Previous work has demonstrated potential associations between infectious disease epidemics/pandemics and mental-health outcomes both historically and today. For example, Honigsbaum and Krishnan [2] described neurological conditions observed after the influenza pandemics of 1889 (possibly H3N8) and 1918 (H1N1). During the 1918 pandemic, Karl A. Menninger studied patients at the Boston Psychopathic Hospital with mental-health symptoms associated with influenza, particularly focusing on patients diagnosed with dementia praecox (now a disused term generally replaced with schizophrenia) [3]. Although Van Der Heide and Coutinho [4] found no effect on asylum hospitalisations in Amsterdam, Mamelund [5] found a sevenfold excess in the number of first-time hospitalised patients with mental diseases associated with the pandemic (i.e. between 1918 and 1923 relative to the average numbers in the 1915–1917 and 1924–1926 periods for Norway). Another condition associated, although somewhat controversially, with the 1918 pandemic was the encephalitis lethargica epidemic between 1917 and the late 1920s, which caused largely somatic or physical effects in adults, notably postencephalitic parkinsonism and paralysis, but also severe psychiatric and behavioural effects in children [6]. Further, Wasserman [7] concluded that the 1918 pandemic – and not other potential factors such as World War I or Prohibition – increased suicide rates, possibly due to reduction in social integration and fear of the epidemic. More recently, Stack and Rockett [8] came to similar conclusions, although both studies have been disputed by Gaddy [9]. Other studies have investigated potential connections between in utero exposure to the 1918 pandemic and health, socio-economic and other outcomes later in life (e.g. Helgertz and Bengtsson [10]).

More recently, work on the COVID-19 pandemic has looked at consequences for those with and without a previous history of psychiatric conditions. For example, Taquet et al. [11] found that a diagnosis of COVID-19 was associated with increased incidence of a first psychiatric diagnosis in the following 14–90 days for patients with no previous psychiatric history. Worsening of conditions such as depression and post-traumatic stress disorder (PTSD) has been observed among psychiatric patients, again possibly related to, among other factors, reduced interaction with family and friends and a feeling of no control [12]. Further, highlighting the role of factors other than direct infection, a cross-sectional sample of Canadian adult men using an eHealth depression resource reported negative mental-health effects of the COVID-19 pandemic and physical distancing measures, as well as concerns related to their financial, living and relationship situations [13]. Additionally, research on the severe acute respiratory syndrome coronavirus-1 (SARS) epidemic of 2003 showed, for example, that the prevalence of post-SARS psychiatric disorders was approximately one-third in a cohort of 90 participants 30 months after infection [14]. Other studies have focused on smaller epidemics and infectious diseases in general, such as research on influenza-associated neurological complications in children, which tend to be relatively short term or transient (e.g. Wang et al. [15] and Tzang et al. [16]).

Overall, the body of work on this topic addresses a variety of infectious diseases and mental-health conditions, but often for specific groups (e.g. children, psychiatric patients) and with relatively short-term or cross-sectional data and, in the case of historical pandemics particularly, small samples or even anecdotal data. Therefore, there is a clear need for systematic reviews to consolidate and make sense of different findings. Several narrative and systematic reviews, some with meta-analyses, have been conducted since the onset of the COVID-19 pandemic. However, these studies also have many of the same limitations, such as relatively narrow foci on specific populations, mental-health conditions or potential causative factors. For example, Tappenden and Tomar [17] focused on the consequences of social isolation among older people, while Samji et al. [18] and Meherali et al. [19] investigated mental-health impacts on children and adolescents. Further, Neelam et al. [20] and Sergeant et al. [21] reviewed studies on mental health of people with pre-existing mental illness, and Serrano-Ripoll et al. [22] and Zaçe et al. [23] considered the mental health of health-care workers. Although some reviews have specifically considered post-viral and long-term mental-health effects of COVID-19 [24] and longitudinal analyses before and during the pandemic [25], the ongoing nature and relatively short duration since the onset of the COVID-19 pandemic necessarily restricts possible conclusions about longer-term or post-pandemic effects. Systematic reviews that have included other, older epidemics or pandemics include Yuan et al. [26], who reviewed 88 studies about the prevalence of PTSD following pandemics, including Ebola, Zika, Nipah, MERS and polio, among others. Almost all the studies evaluated mental-health outcomes within one year of the relevant outbreak or epidemic, and none did so more than two years after. Zürcher et al. [27] found that prevalence of mental-health problems following confirmed or suspected infection with 2003 SARS, 2009–2010 swine flu (H1N1), Ebola or COVID-19, among other diseases, decreased over time but were still substantial in post-illness stages, defined as longer than three months. In such examples, the focus on a specific mental-health outcome [26] and pandemic-related mental-health consequences only among those who were infected [27], as well as including results for many varied epidemic diseases, may limit or confound the results.

The protocol for a forthcoming systematic review described here thus contributes to the literature in several ways. First, we consider long-term effects of historical influenza pandemics as far back as the end of the 19th century (1889), allowing for potential identification or evaluation of substantially long-lasting and/or post-pandemic consequences. Second, we recognise that pandemics have wide-ranging effects on all aspects of society and so do not restrict our analyses based on population type or the assumed cause of mental-health consequences (e.g. direct infection). Similarly, we consider a wide range of potential mental-health outcomes rather than only certain diagnoses and/or related ICD codes. Finally, we limit our review to pandemics believed or known to have been caused by strains of influenza to minimise confounding aspects of different kinds of diseases, albeit there has been some work (e.g. Brüssow and Brüssow [28]) arguing the ‘Russian flu’ of 1889–1890 was caused by a coronavirus rather than influenza. The protocol and forthcoming systematic review will not include COVID-19 because COVID-19 is caused by the SARS-CoV-2 coronavirus, and as noted, the ongoing nature and relatively short duration since the onset of the COVID-19 pandemic limits possible conclusions about longer-term or post-pandemic effects.

## Methods

We will conduct a systematic review and narrative synthesis. The following protocol is reported using guidance from the Preferred Reporting Items for Systematic Reviews and Meta-Analyses Protocols (PRISMA-P 2015) [29] (see Supplemental Material). The protocol has been registered with the International Prospective Register of Systematic Reviews (PROSPERO) with the registration number CRD42021253307. The planned systematic review resulting from the protocol also will be reported following PRISMA.

### Eligibility criteria

The systematic review will synthesise findings from studies that investigate the association between influenza pandemics and long-term mental-health sequelae from direct infection or related indirect circumstances. Studies addressing pandemic diseases besides influenza, seasonal influenza only, both seasonal and pandemic influenza without distinguishing between them, or potential mental-health side effects of vaccines or treatments will not be eligible. Only English-language studies will be included, although studies investigating all regions and countries are eligible. The study period will encompass 1889–2009 to account for the five historical influenza pandemics of ‘Russian flu’ of 1889–1890 (possibly H3N8), the ‘Spanish flu’ of 1918–1920 (H1N1), ‘Asian flu’ of 1957–1958 (H2N2), ‘Hong-Kong flu’ of 1968–1970 (H3N2), and ‘swine flu’ of 2009–2010 (H1N1). Following the PICO framework, populations (P) may include both those with and without pre-existing mental-health symptoms or conditions prior to the associated pandemic. Intervention (I) is exposure of the population or participants to an influenza pandemic during the study period. Comparators or controls (C) are not relevant for this review. The review will address outcomes (O) of mental-health morbidity and related impacts, including social or economic impact, institutionalisation and/or death. In general, research using any study design will be eligible for inclusion, except for case studies that address the symptoms or diagnoses of individuals, commentary or review pieces without original data, and articles on policy and/or social justice issues. Further, although eligible articles may discuss both mental and physical health effects (e.g. lung damage, heart and kidney disease, hearing loss or deafness), studies will be ineligible if they address long-term physical effects only. Studies linking late-in-life mental health with foetal/early exposure, and studies of mental-health symptoms during influenza-like illness that resolve after acute infection (i.e. short-term effects), will also be ineligible.

### Information sources and search strategy

The search strategy was developed and piloted in collaboration with research librarians at Oslo Metropolitan University to ensure sensitivity and precision. Searches took place between January and February 2021 and were conducted in Embase, MEDLINE, PsycINFO, CINAHL, Web of Science, Academic Search Ultimate, ASSIA and Google Scholar. The final search strategy comprised two elements and an extra search: influenza pandemic/epidemics, mental-health outcomes and long-term effects and pandemics (in the title only). Mental-health keywords included general terms (e.g. mental disorders) and specific conditions (e.g. schizophrenia). Pandemic keywords included specific influenza viruses, pandemic years and colloquial names (e.g. ‘Spanish flu’). Duration-related keywords included ‘long term’, ‘persistent’, ‘chronic’ and so on. The search elements with related terms and synonyms were combined within each database. Neither language nor publication date restrictions were applied during the search. The Supplemental Material provides an example of the search strategy for one database.

### Data management

All eligible studies from all relevant databases (*N*=8190) were imported to EndNote, and any duplicates were removed. These results were then imported into the screening program Covidence, and further duplicates were removed, leaving 4428 articles. Following screening of the titles and abstracts, we will obtain full-text versions of the studies, which will also be screened in Covidence.

### Selection process

In accordance with the predefined selection criteria, the first two authors (J.D. and B.S.) will independently screen titles and abstracts. After this initial screening, full-text versions will be reviewed for inclusion. Studies over which there is discordance will be reassessed by the same researchers and, if necessary, the third author (S.E.M.) until agreement is reached. A PRISMA diagram will be constructed for transparent documentation of the selection process.

### Data - collection process

The first two researchers will draft a data abstraction form, which will be pilot tested and modified if necessary. The extracted data will be reviewed independently, and any conflicts or concerns will be resolved within the team. Considering the possibility of highly variable outcomes addressed by different studies and the fact that the review will be narrative only, any missing data will simply be noted as absent for relevant individual studies.

### Data items

Extracted data, when applicable, will include: (a) author and publication information, (b) sample and study details (e.g. country or region, pandemic year(s), sample size, source of outcome data, study design), (c) outcomes (see below) and (d) independent variables or contextual details (e.g. presumed cause such as infection, socio-economic effects, bereavement, fear, etc.) and sex, age, education, income and other demographic variables of affected individuals.

### Outcomes and prioritisation

The main outcomes of interest are mental-health morbidity, as well as related effects of pandemics. For morbidity, we will consider outcomes including the mental-health conditions observed, whether they were new diagnoses, when they appeared and how long they lasted, the proportion of the sample affected, and any measures of severity. Related impacts include, for example, whether the affected individuals were registered as disabled or received a pension, institutionalisation (including whether it was voluntary and the type of institution) and/or death (including whether it resulted from suicide or other causes with mental health attributed).

### Risk of bias in individual studies

This narrative review may include studies with any type of research design. Generally, all studies will be evaluated on the details of the sample or data source such as size and representativeness, and the methods or standards used for identifying mental-health outcomes (e.g. diagnosed by doctor, self-reported, etc.). Assessment of quantitative studies will also include evaluation of whether studies controlled for important confounders such as age and sex, the timeframe/length of follow-up and participant attrition, and the selection and application of appropriate statistical methods. Qualitative studies will be appraised based on clarity and appropriateness of the aims, reflexivity of the authors, the research design and methodology, and data collection and analysis. The two reviewers will assess the overall quality of each included study, and any discrepancies will be resolved through discussion.

### Data

#### Synthesis

Results will be synthesised narratively. The descriptive narrative review will include a table of the characteristics of the included studies (e.g. author and year, pandemics studied, region, outcomes, etc.). No meta-analyses of quantitative data will be performed. The narrative review will evaluate the range of presumed causative factors and outcomes overall, as well as consider differences (a) by individual pandemics to account for historical influences, including differences in how mental-health diagnoses may be considered, and (b) for people with and without pre-existing mental-health conditions, as the sampling strategy and outcomes of interest in studies of these groups may differ. We also will assess limitations and gaps to identify future research needs.

#### Meta-biases and confidence in cumulative evidence

No assessment of meta-biases will be performed. By pooling the evaluations of individual studies discussed above, the quality of evidence (low to high) will be assessed for the overall sample, as well as studies associated with different pandemics and methodologies. In the narrative review, conclusions of cumulative quality will be determined by the consistency of the findings, with the assessment downgraded by discrepancies and missing information.

## Discussion

Pandemics have substantial health and social consequences that extend beyond the actual illness or official counts of cases and deaths. These consequences include effects on mental health as the result of, for example, long-term morbidity, bereavement, social isolation and economic losses. Although pandemics are not indiscriminate (e.g. Mamelund and Dimka [30]), a wide range of the population is likely to be affected in some way. To our knowledge, the systematic review described by this protocol is the first on the topic that does not restrict by population at risk or mental-health diagnosis. We also do not limit our review based on the presumed cause of mental-health outcomes (e.g. direct infection). Further, the added value of a historical perspective enables consideration of relatively shorter- and longer-term durations of mental-health consequences. Similarly, the focus on respiratory pandemics with related (presumed) causative pathogens reduces the confounding effects of the type of disease, while comparisons of the included pandemics, taking into account variation in severity and chronological factors, may also give important insights into the persistence or consistency of pandemic consequences over time.

An important limitation of the planned review – and indeed of all reviews that have previously focused on mental-health consequences – is the exclusion of long-term consequences considered to be primarily physiological or somatic in nature. The boundary between mental, emotional and physical health is not distinct, while the correlation or association between them may be considerable, if not always obvious or noted in the literature. Exclusion/inclusion decisions on certain conditions (e.g. encephalitis, sleep disorders) may thus require judgement calls when reaching consensus among the authors, and future work should consider both mental and physical long-term consequences. Additionally, although the eligibility criteria exclude analyses of short-term mental-health effects associated with acute infection only, we do not specify any criterion for how long a condition must last to be deemed a long-term consequence. Again, judgement calls may be necessary when screening articles, and we will address potential findings related to duration in the narrative review.

The results of this review will be submitted for publication in a relevant peer-reviewed journal, as well as presented to academic and non-academic audiences, with awareness of the ethical considerations of proper communication due to mental-health stigma and related concerns. Any amendments made to the protocol will be clearly detailed in those dissemination activities. The results are expected to have important implications for pandemic preparedness and public health policy and practice, including COVID-19 recovery. To truly measure and respond to the full impact of pandemics, long-term morbidity and mortality must also be recognised and counted. This review will thus improve understanding of pandemic-attributable disease, disability and and/or death because of mental-health consequences.

## Supplemental Material

sj-doc-1-sjp-10.1177_14034948231217362 – Supplemental material for Protocol for a systematic review to understand the long-term mental-health effects of influenza pandemics in the pre-COVID-19 era Supplemental material, sj-doc-1-sjp-10.1177_14034948231217362 for Protocol for a systematic review to understand the long-term mental-health effects of influenza pandemics in the pre-COVID-19 era by Jessica L. Dimka, Benjamin M. Schneider and Svenn-Erik Mamelund in Scandinavian Journal of Public Health

sj-docx-1-sjp-10.1177_14034948231217362 – Supplemental material for Protocol for a systematic review to understand the long-term mental-health effects of influenza pandemics in the pre-COVID-19 era Supplemental material, sj-docx-1-sjp-10.1177_14034948231217362 for Protocol for a systematic review to understand the long-term mental-health effects of influenza pandemics in the pre-COVID-19 era by Jessica L. Dimka, Benjamin M. Schneider and Svenn-Erik Mamelund in Scandinavian Journal of Public Health
