# Supplementary material for: Protocol for a systematic review to understand the long-term mental-health effects of influenza pandemics in the pre-COVID-19 era
Source: Scand J Public Health. 2023 Dec 28;52(3):391–6. doi: 10.1177/14034948231217362 (PMC11067412; doi:10.1177/14034948231217362)
Supplement: sj-docx-1-sjp-10.1177_14034948231217362 – Supplemental material for Protocol for a systematic review to understand the long-term mental-health effects of influenza pandemics in the pre-COVID-19 era [file sj-docx-1-sjp-10.1177_14034948231217362.docx]

**Database**: Ovid MEDLINE(R) ALL 1946 to March 09, 2021

**Date**: 11.03.2021

**Number of hits:**  1475

| **#** | **Searches** | **Results** |
| --- | --- | --- |
| 1 | orthomyxoviridae/ or influenzavirus a/ or influenza a virus/ or influenza a virus, h1n1 subtype/ or influenza a virus, h1n2 subtype/ or influenza a virus, h2n2 subtype/ or influenza a virus, h3n2 subtype/ or influenza a virus, h3n8 subtype/ | 50248 |
| 2 | Influenza Pandemic, 1918-1919/ | 170 |
| 3 | Influenza, Human/ | 50969 |
| 4 | (influenza or influenzas or influenzavirus or flu or flus or grippe or H1N1 or H1N2 or H2N2 or H3N2 or H3N8).tw,kw,kf. | 112338 |
| 5 | (PH1N1 or H1N1pdm09 or H1N1p or A?H1N1*).tw,kw,kf. | 1311 |
| 6 | ((Russian or Spanish or Asian or Hong Kong or Mexican) adj4 (pandemic* or epidemic*)).tw,kw,kf. | 927 |
| 7 | (("1889" or 1889-90 or 1889-1890 or 1889-91 or 1889-1891 or 1889-92 or 1889-1892 or 1889-93 or 1889-1893 or 1889-94 or 1889-1894 or "1918" or 1918-19 or 1918-1919 or 1918-20 or 1918-1920 or "1957" or "1958" or 1957-58 or 1957-1958 or "1968" or 1968-69 or 1968-1969 or 1968-70 or 1968-1970 or "1969" or 1969-70 or 1969-1970 or "1970" or "2009" or 2009-10 or 2009-2010) adj7 (pandemic* or epidemic* or outbreak*)).tw,kw,kf. | 9463 |
| 8 | or/1-7 | 122346 |
| 9 | disease outbreaks/ | 82930 |
| 10 | epidemics/ | 11213 |
| 11 | pandemics/ | 52290 |
| 12 | (pandemic* or outbreak* or epidemic*).tw,kw,kf. | 265301 |
| 13 | or/9-12 | 312861 |
| 14 | 8 and 13 | 34665 |
| 15 | Mental Health/ | 42070 |
| 16 | mental disorders/ or exp anxiety disorders/ or exp "bipolar and related disorders"/ or "disruptive, impulse control, and conduct disorders"/ or exp dissociative disorders/ or exp "feeding and eating disorders"/ or exp mood disorders/ or exp neurocognitive disorders/ or exp neurodevelopmental disorders/ or exp personality disorders/ or exp "schizophrenia spectrum and other psychotic disorders"/ or exp somatoform disorders/ or exp "trauma and stressor related disorders"/ or exp sleep wake disorders/ | 1055495 |
| 17 | neurasthenia/ | 1358 |
| 18 | catatonia/ | 2539 |
| 19 | mental fatigue/ | 1618 |
| 20 | depression/ | 125116 |
| 21 | Anxiety/ | 85470 |
| 22 | exp suicide/ | 64982 |
| 23 | Fatigue Syndrome, Chronic/ | 5587 |
| 24 | Encephalitis/ | 18876 |
| 25 | exp Parkinsonian Disorders/ | 83124 |
| 26 | Fatigue/ | 29689 |
| 27 | sleepiness/ | 425 |
| 28 | Psychological Distress/ | 1472 |
| 29 | exp Amnesia/ | 8524 |
| 30 | Memory Disorders/ | 21709 |
| 31 | Neurologic Manifestations/ | 7323 |
| 32 | exp Neurobehavioral Manifestations/ | 283311 |
| 33 | Headache/ | 28318 |
| 34 | exp Cognition/ | 167143 |
| 35 | Attention/ | 79659 |
| 36 | ((mental* or psychologic* or psychiatric* or psychic* or psychopatholog* or neuropsycholog* or neuropsychiatr* or neurotic* or neurologic* or cognit* or concentration* or attention*) adj3 (illness* or disorder* or health* or syndrome* or symptom* or status or state* or impairment* or complication* or admission* or distress* or retard* or handicap* or problem* or dysfunction*)).tw,kw,kf. | 713541 |
| 37 | (exhaustion or tiredness or sleep* or memory loss or amnesia or confusion* or fatigue or myalgic encephalomyelitis or neurastheni* or anxi* or depression* or depressive* or depressed or melancholi* or mania* or manic* or catatoni* or letharg* or delirium or bipolar or dementia or schizophren* or suicid* or post-traumatic stress* or ptsd or narcolep* or (encephaliti* adj4 lethargic*) or parkinson* or paralysis agitans or neuromyastheni* or systemic exertion* intolerance disease* or cfs or dysthymi* or dysphori* or alzheimer* or schizoaffect* or agoraphobi* or von Economo or botulism or toxic ophthalmoplegia or epidemic stupor or acute polioencephalitis or Heine-Medin disease or bulbar paralysis or hystero-epilep* or (cloud* adj4 conscious*) or ((brain or mental) adj4 fog*) or headache* or head ache*).tw,kw,kf. | 1512988 |
| 38 | ((long term* or longterm* or long period* or long haul* or longhaul* or long run* or sequelae* or ((post or postacute* or persistent*) adj4 (illness* or syndrome* or prolonged or symptom*)) or (persistent adj3 (infecti* or symptom* or syndrome*)) or (chronic adj3 (complication* or infect* or symptom* or syndrome* or illness*))) adj6 (pandemic* or epidemic* or outbreak*)).ti. | 321 |
| 39 | or/15-38 | 2656913 |
| 40 | (14 and 39) not ((exp animal/ or exp invertebrate/ or animal experiment/ or animal model/ or exp plant/ or exp fungus/) not exp human/) | 1475 |
